# Association of male sleep quality with semen parameters and pregnancy outcomes in infertile couple

**DOI:** 10.1186/s12610-025-00287-w

**Published:** 2025-10-01

**Authors:** Rong Zhou, Ming Zhang, Caiyun Ge, Yao Xiong, Mei Wang, Kejia Wu, Yuanzhen Zhang

**Affiliations:** 1 Reproductive Medicine Center, Zhongnan Hospital of Wuhan University, Wuhan, Hubei 430071 People’s Republic of China; 2 Clinical Research Center for Prenatal Diagnosis and Birth Health of Hubei Province, Wuhan, China; 3 Clinical Research Center for Reproductive Science and Birth Health of Wuhan, Wuhan, China

**Keywords:** Male sleep quality, Male infertility, Semen parameters, Hormones profiles, Pregnancy outcomes, Pittsburgh Sleep Quality Index, Qualité du Sommeil masculin, Infertilité masculine, Paramètres du Sperme, Profils hormonaux, Issues de la Grossesse, Indice de Qualité du Sommeil de Pittsburgh

## Abstract

**Background:**

Sleep quality has been increasingly recognized as an important determinant of overall health, yet its influence on male fertility remains underexplored. This study investigated the association between male sleep quality and reproductive outcomes, including semen characteristics, hormone profiles, and partner pregnancy success, in infertile couples.

**Results:**

A total of 727 male partners from infertile couples were evaluated between October 2023 and February 2025. Sleep quality was assessed using Pittsburgh Sleep Quality Index and categorized as good or poor. Poor sleep quality was reported in 75.1 percent of participants. Men with poor sleep quality showed significantly lower sperm concentration (β = -1.39, 95% confidence interval = -2.11 to -0.67, *p* < 0.001), reduced progressive motility (β = -1.25, 95% confidence interval = -1.61 to -0.88, *p* < 0.001), and decreased total motility compared with those reporting good sleep. No significant associations were observed between sleep quality and hormone concentrations, including follicle-stimulating hormone, luteinizing hormone, estradiol, prolactin, and testosterone. Poor male sleep quality was also linked to a lower probability of achieving clinical pregnancy (odds ratio = 4.67, 95% confidence interval = 3.08 to 7.09, *p* < 0.0001).

**Conclusions:**

Poor male sleep quality is associated with impaired semen quality and reduced chances of pregnancy in couples with infertility. These findings highlight the potential value of improving sleep as a modifiable factor to enhance male reproductive health and fertility outcomes.

**Supplementary Information:**

The online version contains supplementary material available at 10.1186/s12610-025-00287-w.

## Résumé

**Contexte:**

La qualité du sommeil est de plus en plus reconnue comme un déterminant important de la santé globale, mais son influence sur la fertilité masculine reste sous-explorée. Cette étude a examiné l’association entre la qualité du sommeil masculin et les résultats reproductifs, y compris les caractéristiques du sperme, les profils hormonaux et le succès de la grossesse du partenaire, chez les couples infertiles.

**Résultats:**

Un total de 727 partenaires masculins issus de couples infertiles ont été évalués entre octobre 2023 et février 2025. La qualité du sommeil a été évaluée à l’aide de l’indice de qualité du sommeil de Pittsburgh et classée comme bonne ou mauvaise. Une mauvaise qualité de sommeil a été signalée chez 75,1% des participants. Les hommes ayant une mauvaise qualité de sommeil présentaient une concentration de spermatozoïdes significativement plus faible (β = −1,39, intervalle de confiance à 95% = −2,11 à −0,67, *p* < 0,001), une motilité progressive réduite (β = −1,25, intervalle de confiance à 95% = −1,61 à −0,88, *p* < 0,001) et une motilité totale réduite par rapport à ceux signalant un bon sommeil. Aucune association significative n’a été observée entre la qualité du sommeil et les concentrations d’hormones, y compris l’hormone folliculo-stimulante, l’hormone lutéinisante, l’œstradiol, la prolactine et la testostérone. Une mauvaise qualité de sommeil chez les hommes était également liée à une probabilité plus faible d’obtenir une grossesse clinique (OR = 4,67, intervalle de confiance à 95% = 3,08 à 7,09, *p* < 0,0001).

**Conclusions:**

Une mauvaise qualité de sommeil masculin est associée à une altération de la qualité du sperme et à une réduction des chances de grossesse chez les couples souffrant d’infertilité. Ces résultats soulignent la valeur potentielle de l’amélioration du sommeil en tant que facteur modifiable pour améliorer la santé reproductive et la fertilité masculine.

## Background

Sleep and wakefulness are regulated by intrinsic neural circuits and modulated by the body's internal circadian system. These biological clocks operate through self-sustained, rhythmic fluctuations in physiological activity, following an approximately 24-h cycle [1]. Sleep is an essential process in mammals, playing a crucial role in memory consolidation, homeostasis regulation, and immune function. Adequate rest is widely recognized as vital for overall well-being, and substantial evidence links insufficient sleep to declining health status [2]. According to the 2022 China Sleep Research Report released by the Chinese Sleep Research Society, the average nightly sleep duration among the Chinese populace has decreased by approximately 1.5 h over the past decade. Sleep disorders have become increasingly prevalent over time, emerging as a major health concern frequently addressed in clinical practice [3]. In the general adult population, over 50% report occasional sleep disturbances, and approximately 15–20% experience chronic sleep problems [4]. Beyond its well-established effects on general health, growing evidence suggests that inadequate or poorly timed sleep may also impair male fertility. Globally, more than 30 million men are affected by infertility, especially in Eastern Europe and Africa, due to factors including testicular dysfunction, endocrine disorders, unhealthy lifestyles, and aging [5, 6]. Studies indicate that insufficient rest and suboptimal sleep patterns may also adversely affect sperm quality and reproductive outcomes [7–9].

Sleep is a vital physiological process that plays a critical role in hormonal regulation and reproductive health. Growing evidence suggests that both the quantity and quality of sleep may influence male fertility, particularly semen parameters. Experimental studies in animals have demonstrated that sleep deprivation can damage sperm acrosomal integrity and DNA integrity in mice [10], cause irreversible morphological abnormalities in sperm [11]. Similarly, cohort studies in humans have shown that complete sleep deprivation significantly reduces serum testosterone levels in men [12]. However, findings across studies remain inconsistent. For example, some research indicates that both prolonged and insufficient rest periods are linked to irregular semen parameters [9, 13], while other studies have found no link between extended sleep duration and poor sperm quality [14]. A population-based cohort study reported that sleep duration and sleep onset time were associated with sperm quality in men seeking fertility treatment [15], whereas another study reported a positive correlation between sleep duration and sperm concentration; sleep latency was negatively correlated with total sperm count among men attending infertility clinics [16]. Given these conflicting findings, the relationship between sleep duration, male sleep quality, and male fertility remains inconclusive. Discrepancies may be due to differences in inclusion criteria, statistical approaches, and the geographic characteristics of study populations. Despite emerging evidence, two major research gaps persist. First, few studies have comprehensively examined the association between male sleep quality and male fertility. Second, little is known about whether male sleep quality influences pregnancy outcomes, particularly in the context of cultural, lifestyle, and environmental factors that may shape reproductive health.

To address these gaps, this study uses the Pittsburgh Sleep Quality Index (PSQI), a validated and widely used questionnaire in which higher scores indicate poorer sleep quality. Although extensively applied internationally, the PSQI has seen limited use in China [17–19]. We hypothesize that poor male sleep quality is associated with reduced semen quality and lower pregnancy success rates in infertile couples.

## Patients and methods

### Participants and setting

Participants were recruited from the Reproductive Medicine Center, Zhongnan Hospital of Wuhan University. Wuhan, China from October 2023 to February 2025.The sample size was estimated using G*Power 3.1.9.7. The minimum sample size was calculated to be 234, based on medium effect size of 0.15, significance level α of 0.05, power (1-β) of 0.95 [20]. A total of 281 participants were requested concerning 20% rate of loss of follow-up.

### Ethical consideration

The study was approved by the ethics committee of of Zhongnan Hospital of Wuhan University (No.2024071 K). All participants signed the informed consent and were told that their participation was voluntary,confidential and no potential injuries and risks.

### Data collection

The participants' basic demographic information, including the male partner's age, weight, body mass index (BMI), educational background, and smoking history, as well as the duration and type of infertility, the female partner’s medication regimen, and pregnancy outcomes, was obtained from the hospital's electronic medical record system. Patient data were anonymized and kept confidential during the analysis. After hospital admission, participants completed the PSQI questionnaire, underwent semen quality assessment, and sex hormone testing. Cases with duplicate entries, incomplete responses, medication use during a natural cycle, non-infertility diagnoses, or missing key baseline variables were excluded from the analysis.

### Measurement of male sleep quality

The PSQI questionnaire was used to assess male sleep quality. This self-reported survey consists of 19 items reflecting the respondent’s sleep quality over the past month. The questionnaire includes seven components: subjective sleep quality, sleep latency, sleep duration, habitual sleep efficiency, sleep disturbances, use of sleeping medication, and daytime dysfunction. Each component is scored on a scale of 0 to 3, where "0" indicates no difficulty, "1" indicates mild difficulty, "2" indicates moderate difficulty, and "3" indicates severe difficulty. The total PSQI score is obtained by summing the scores of all components, ranging from 0 to 21, with higher scores indicating poorer male sleep quality. Studies have reported that the Cronbach’s α of PSQI in China is 0.757 [21]. Based on previous literature [17, 22], we classified participants into two male sleep quality groups: Good sleep: PSQI ≤ 5 and Poor sleep: PSQI > 5. The detailed description of PSQI is provided in Supplementary materials.

### Semen analysis

According to medical records, participants had abstained from ejaculation for 2–7 days before collecting semen samples through masturbation. The samples were collected in a sterile plastic container and delivered to the laboratory within 30 min after ejaculation. After liquefaction at 37 °C, the samples were analyzed for sperm concentration, sperm motility, sperm morphology, and DNA fragmentation index (DFI). All experiments were conducted under standardized conditions following world health organization (WHO) recommendations [23]. Sperm motility was categorized into progressive motility (PR) and non-progressive motility (NP). PR = total PR/total sperm × 100% and NP = total NP/total sperm × 100%. Total motility = PR + NP, DFI = (Number of sperm with DNA damage/Total number of sperm analyzed) × 100%.

### Hormone profiles tests

Hormone profiles testing was typically conducted through venous blood collection, with the recommended sampling time being in the morning on an empty stomach (8:00–10:00 AM). After centrifugation to separate the serum,. reproductive hormones including follicle-stimulating hormone (FSH), luteinizing hormone (LH), estradiol (E_2_), testosterone (T), and prolactin (PRL) were measured at the clinical laboratory of Zhongnan Hospital of Wuhan University.

### Pregnancy outcome assessment

Pregnancy outcomes were assessed based on biological, imaging, and clinical evidence collected up to the data collection point. Only couples with complete pregnancy outcome data were included in the analysis. Clinical pregnancy was defined by ultrasound visualization of an intrauterine gestational sac, yolk sac, and fetal heartbeat, typically observed at 5–6 weeks of gestation. Participants lacking follow-up pregnancy outcome data were excluded from the analysis.

### Statistical analysis

The normality of the data was determined via the Kolmogorov–Smirnov test, and the homogeneity of variances was checked through graphical inspection of residuals. For continuous variables, if they followed a normal or approximately normal distribution, they were presented as mean ± standard deviation (SD), and independent sample t-tests were used for group comparisons. If the data followed a skewed distribution, they were expressed as median (25–75 percentiles), and Mann–Whitney U tests were applied. For categorical variables, data were presented as frequency (percentage), and Chi-square tests or Fisher’s exact tests were used for comparisons. Pregnancy status was used as a binary outcome variable, and good or poor sleep was used as the independent variable. Both univariable and multivariable logistic regression analyses were performed to explore the association between male sleep quality and pregnancy outcomes, adjusting for demographic and clinical characteristics. Additionally, PSQI subscale scores and total score were treated as continuous variables to analyze their association with pregnancy outcomes. Pregnancy failure was considered as the positive event. The associations were reported as odds ratios (ORs) with 95% confidence intervals (95% CIs).

The primary outcome of interest was clinical pregnancy. Secondary outcomes included semen parameters (sperm concentration, progressive motility, total motility, DFI) and serum hormone profiles (FSH, LH, E2, PRL, and testosterone).

We also used univariable and multivariable linear regression analyses to investigate the relationship between male sleep quality, semen parameters, and hormone profiles. Male sleep quality was analyzed using the PSQI total score. Two regression models were established: the first model included only male sleep quality without adjusting for any covariates, while the second model further adjusted for potential confounders related to semen parameters, including age, BMI, and smoking. Additionally, we calculated the Spearman correlation coefficients between the PSQI total score and all semen parameters. Statistical analyses were conducted using SAS software, version 9.4 TS1M6 (SAS Institute Inc.,), and a two-sided *p*-value < 0.05 was considered statistically significant.

## Results

### Participant characteristics

**Fig. 1:**
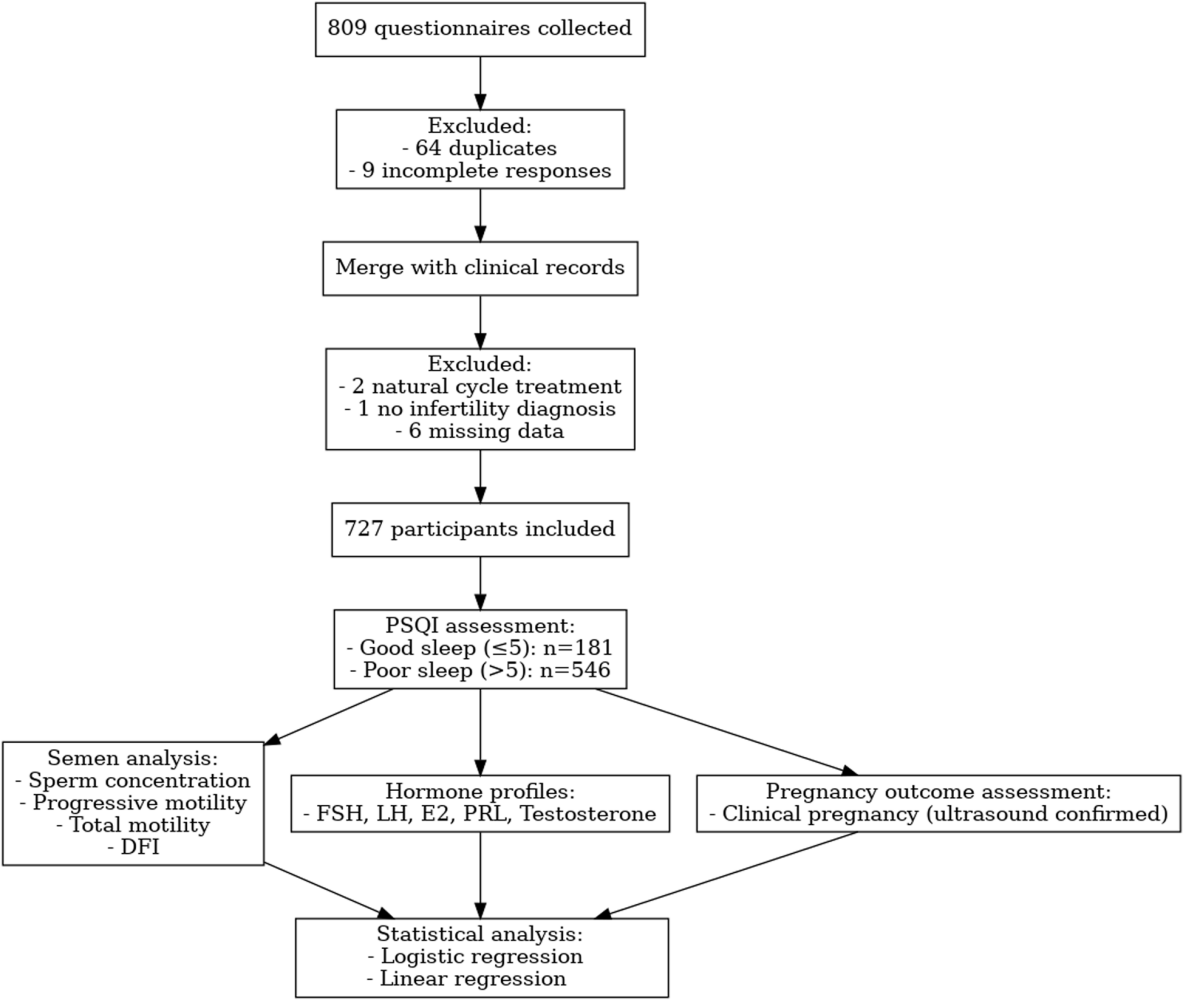
Flowchart of the present study. DFI, DNA fragmentation index; E2, estradiol; FSH, follicle-stimulating hormone; LH, luteinizing hormone; PRL, prolactin; PSQI, Pittsburgh Sleep Quality Index

A total of 809 sleep questionnaires were collected. After removing 64 duplicates and 9 incomplete responses, the data were merged with clinical records. An additional 9 participants were excluded due to natural cycle treatment (*n* = 2), lack of an infertility diagnosis (*n* = 1), or missing baseline/clinical variables such as male smoking history, BMI, infertility duration, and medication regimen (*n* = 6). In total, 727 male subjects were included in the final analysis. The study is summarised in the flowchart shown in Fig. 1. The demographic and reproductive profiles of the analyzed cohort are presented in Table 1. The age of subjects varied between 23 and 60 years. Based on PSQI total scores, individuals were classified into good sleep (PSQI ≤ 5, *n* = 181) and poor sleep groups (PSQI > 5, *n* = 546). Among infertile couples, 75.1% of men were classified as having poor Sleep. No significant differences were observed between the good and poor sleep groups in age, BMI, or educational background (all *P* > 0.05). However, the good sleep group had a higher proportion of non-smokers (70.17% vs. 60.62%, *P* = 0.021). Most participants received an antagonist protocol, with no group difference (*P* = 0.172). Pregnancy success was significantly higher in the good sleep group (81.22% vs. 49.08%,* P* < 0.0001).

**Table 1:** Demographic and reproductive characteristics of study populations

Variables	Total (*n* = 727)	Good sleep	Poor sleep	*P*
		(*n* = 181)	(*n* = 546)	
Duration of infertility (year), median (25–75 percentiles)	2 (1,4)	2 (1,3)	2 (1,4)	0.105
Weight (kg) (mean ± SD)	76.15 ± 11.93	76.43 ± 13.31	76.06 ± 11.44	0.717
BMI (kg/m^2^) (mean ± SD)	25.41 ± 3.47	25.69 ± 4.00	25.32 ± 3.27	0.214
Education, n (%)				0.66
Primary school/Junior high school	121 (16.64)	31 (17.13)	90 (16.48)	
High school/Vocational school	112 (15.41)	23 (12.71)	89 (16.30)	
Bachelor's/Associate degree	419 (57.63)	106 (58.56)	313 (57.33)	
Master's degree/Doctorate (Ph.D.)	75 (10.32)	21 (11.60)	54 (9.89)	
Smoking, n (%)				0.021
No	458 (63)	127 (70.17)	331 (60.62)	
Yes	269 (37)	54 (29.83)	215 (39.38)	
Medication regimen, n (%)				0.172
PPOS	141 (19.39)	35 (19.34)	106 (19.41)	
Long protocol of ovarian stimulation	17 (2.34)	8 (4.42)	9 (1.65)	
Long-acting long protocol	26 (3.58)	7 (3.87)	19 (3.48)	
Luteal phase ovarian stimulation protocol	18 (2.48)	2 (1.10)	16 (2.93)	
Antagonist protocol	525 (72.21)	129 (71.27)	396 (72.53)	
Infertility type, n (%)				0.353
Secondary	388 (53.37)	102 (56.35)	286 (52.38)	
Primary	339 (46.63)	79 (43.65)	260 (47.62)	
Pregnancy outcomes				< 0.0001
Non-Pregnant	312 (42.92)	34 (18.78)	278 (50.92)	
Pregnant	415 (57.08)	147 (81.22)	268 (49.08)	

The duration of infertility was obtained from electronic medical records, where physicians recorded the information based on the clinical definition of infertility during patient consultations

*BMI* body mass index, *PPOS* progestin-primed ovarian stimulation, *SD* standard deviation

### Male sleep quality assessment among male participants

Total PSQI scores and the scores of its seven components for all participants, as well as for pregnant and non-pregnant groups, are presented in Table 2. In the overall cohort, the median score for subjective sleep quality was 2, indicating moderate difficulty in subjective sleep quality. Similarly, sleep disturbances and daytime dysfunction were also rated as moderate difficulty. In contrast, sleep latency and habitual sleep efficiency were classified as mild difficulty, while sleep duration and use of sleeping medication were rated as no difficulty.

**Table 2:** Pittsburgh Sleep Quality Index scores

Variables	Total (*n* = 727)	Non-pregnant (*n* = 312)	Pregnant (*n* = 415)
Subjective sleep quality	2 (1,2)	2 (2,2)	1 (1,2)
Sleep latency	1 (1,2)	1 (0,2)	1 (1,2)
Sleep duration	0 (0,1)	0 (0,1)	1 (0,1)
Habitual sleep efficiency	1 (0,1)	1 (0,2)	1 (0,1)
Sleep disturbances	2 (1,2)	2 (1,2)	1 (1,2)
Use of sleeping medication	0 (0,1)	0 (0,1)	0 (0,0)
Daytime dysfunction	2 (1,2)	2 (1,2)	1 (1,2)
Overall score	8 (6,9)	8 (7,10)	7 (5,9)

Data are presented as median with interquartile range

### Association between male sleep quality and pregnancy outcome

Additional regression analysis findings (Table 3) revealed a strong correlation between overall PSQI scores and reproductive success rates. In the unadjusted logistic regression model, the poor sleep group had a significantly lower pregnancy success rate compared with the good sleep group (OR = 4.46, 95% CI: 2.97, 6.72, *P* < 0.0001). After adjusting for male age, BMI, education level, smoking status, duration of infertility, medication regimen, and infertility type, the association remained significant (OR = 4.67, 95% CI: 3.08, 7.09, *P* < 0.0001). When treating PSQI total score as a continuous variable, each one-point increase in PSQI was associated with a 20% increase in the likelihood of pregnancy failure (OR = 1.19, 95%CI:1.14, 1.27, *P* < 0.0001), indicating that poorer overall male sleep quality may negatively impact pregnancy outcomes.

**Table 3:** Analysis of the association between male sleep quality and pregnancy outcomes

		OR (95%CI)	P	Adjusted OR^a^ (95%CI)	*P*
Sleep quality	Good sleep	Ref	-	Ref	-
	Poor sleep	4.46 (2.97,6.72)	< 0.0001	4.67(3.08,7.09)	< 0.0001
Overall PSQI score	-	1.20(1.13,1.26)	< 0.0001	1.20(1.14,1.27)	< 0.0001
Subjective sleep quality		3.43(2.65,4.44)	< 0.0001	3.60(2.76,4.69)	< 0.0001
Sleep latency		0.94(0.80,1.11)	0.475	0.95(0.80,1.12)	0.5417
Sleep duration		0.93(0.76,1.14)	0.4595	0.95(0.77,1.17)	0.634
Habitual sleep efficiency		1.23(1.06,1.42)	0.0052	1.25(1.08,1.46)	0.0029
Sleep disturbances		2.89(2.20,3.81)	< 0.0001	2.91(2.20,3.86)	< 0.0001
Use of sleeping medication		1.93(1.50,2.49)	< 0.0001	1.92(1.49,2.48)	< 0.0001
Daytime Dysfunction		1.49(1.27,1.75)	< 0.0001	1.50(1.27,1.77)	< 0.0001

95%CI, 95% confidence interval; OR, odds ratio

^a^Regression coefficients were adjusted for male age, male body mass index, male education level, male smoking status, duration of infertility, medication regimen and type of infertility

Further analysis of individual PSQI components and pregnancy outcomes, after adjusting for covariates, showed that the following PSQI components were significantly associated with pregnancy outcomes: 1-subjective sleep quality: OR = 3.60, 95%CI:2.76, 4.69, *P* < 0.0001; 4-habitual sleep efficiency: OR = 1.25, 95% CI: 1.08, 1.46, *P* = 0.0029; 5-sleep disturbances: OR = 2.91, 95% CI: 2.20, 3.86, *P* < 0.0001; 6-use of sleeping medication: OR = 1.92, 95% CI: 1.49, 2.48, *P* < 0.0001; daytime dysfunction: OR = 1.50, 95%CI:1.27, 1.77, *P* < 0.0001. Among them, subjective sleep quality and sleep disturbances may be key factors influencing pregnancy success. In contrast, sleep latency (OR = 0.95, 95% CI: 0.80, 1.12, *P* = 0.5417) and sleep duration (OR = 0.95, 95% CI:0.77, 1.17, *P* = 0.634) were not significantly associated with pregnancy outcomes.

### Association between male sleep quality and semen parameters, as well as hormone profiles

**Fig. 2:**
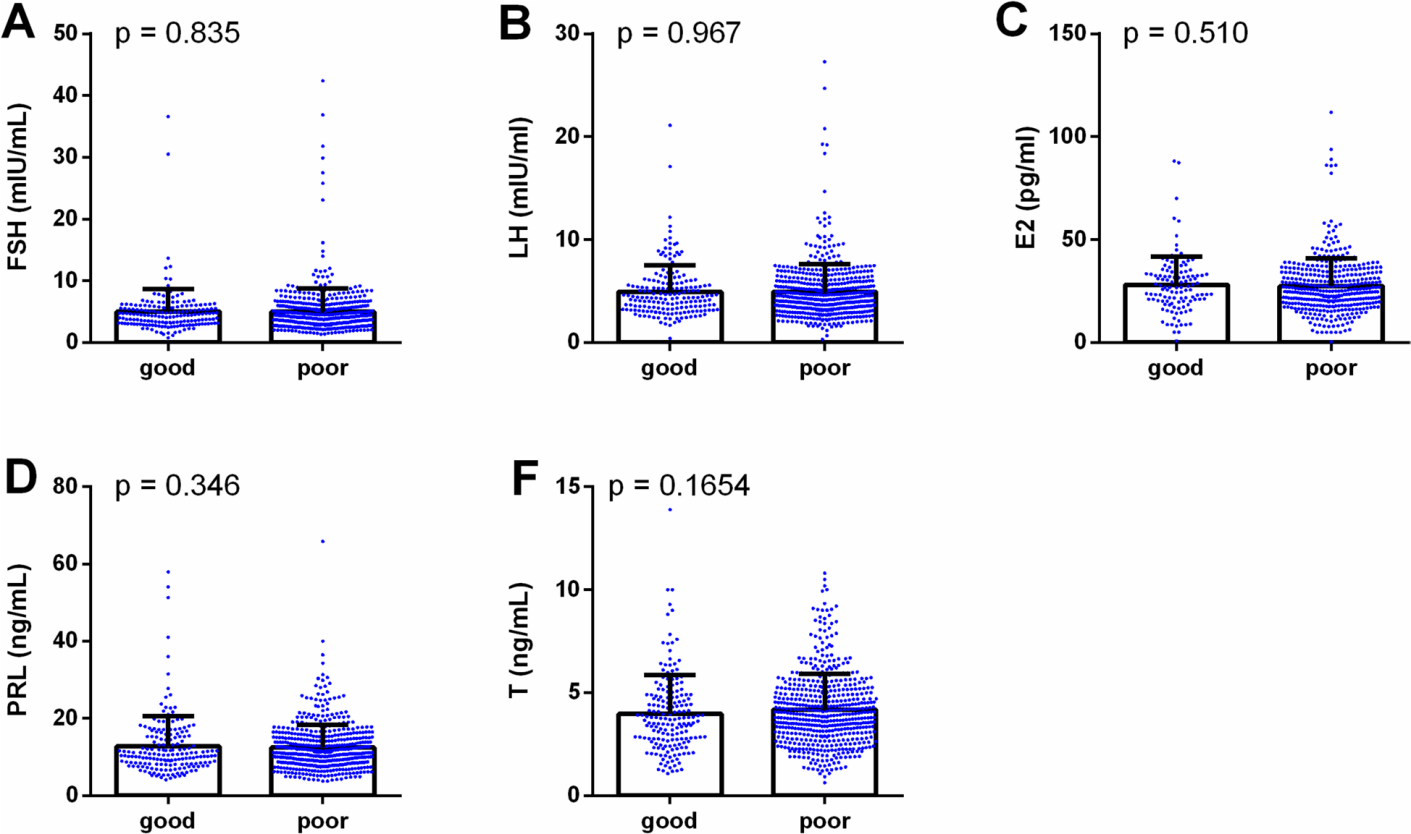
Comparison of hormone profiles between good (*n* = 181) and poor sleep groups (*n* = 546). Bar plots show the distribution of follicle-stimulating hormone (FSH) (**A**), luteinizing hormone (LH) (**B**), estradiol (E2) (**C**), prolactin (PRL) (**D**), and testosterone (T) (**E**) levels in participants with good sleep (PSQI ≤ 5) and poor sleep (PSQI > 5). No significant differences were observed between the two groups

**Fig. 3:**
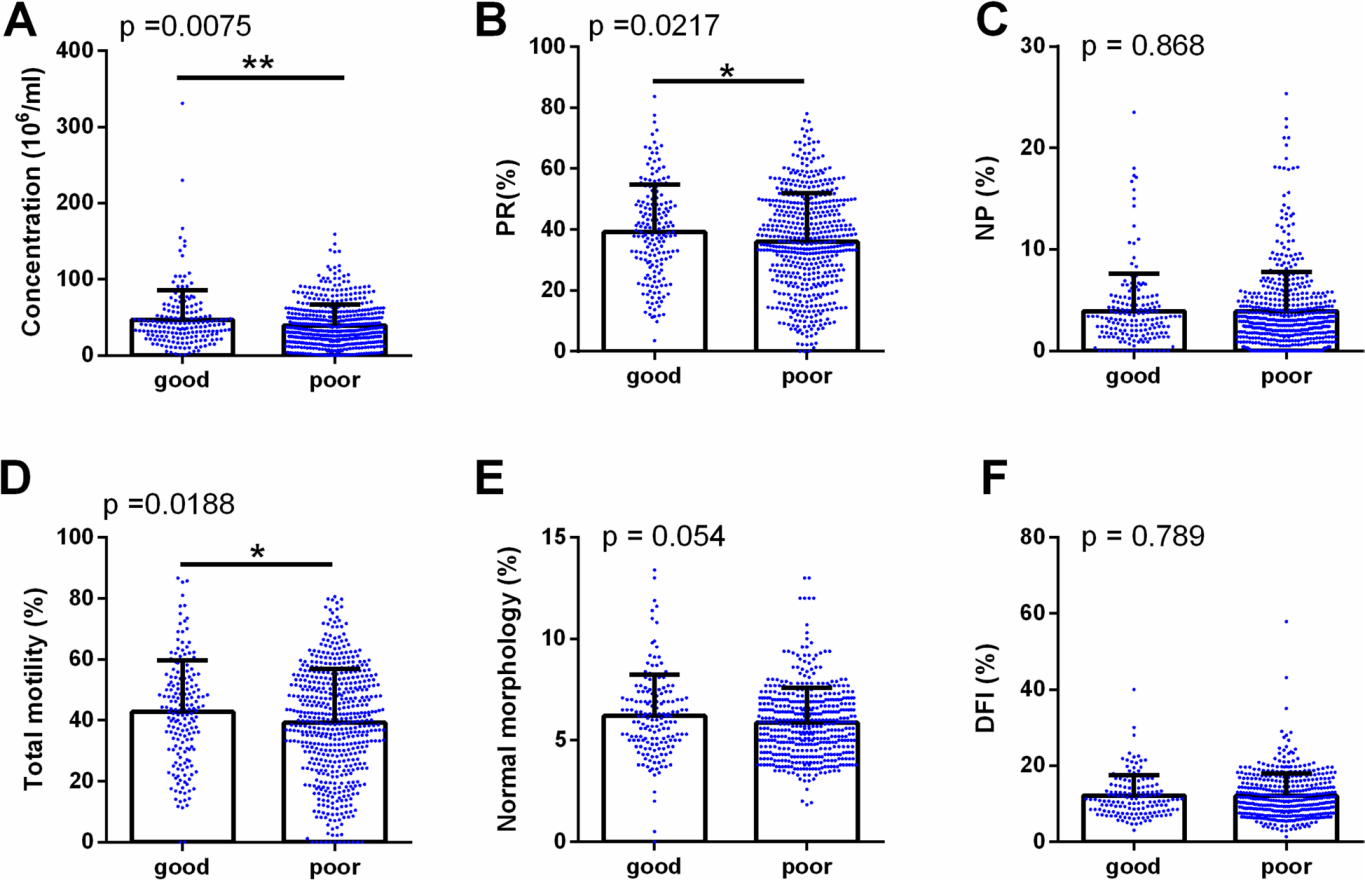
Comparison of semen parameters between good (*n* = 181) and poor sleep groups (*n* = 546). Bar plots illustrate the differences in sperm concentration (**A**), progressive motility (PR%) (**B**), non-progressive motility (NP%) (**C**), total motility (**D**), normal morphology (**E**), and DNA fragmentation index (DFI%) (**F**) between good sleep and poor sleep groups. Significant reductions in sperm concentration, PR%, and total motility were observed in the poor sleep group (*p < 0.05 and **p < 0.01). Other parameters did not show significant differences

The figures illustrate the impact of male sleep quality on hormone profiles and semen parameters. Figure 2 shows no differences in hormone profiles (FSH, LH, E2, PRL, and T) between good and poor sleep groups, suggesting that male sleep quality has a minimal effect on hormone secretion. However, Fig. 3 reveals differences in semen parameters, with lower sperm concentration, PR, and total motility in the poor sleep group. Other parameters, such as NP, normal morphology, and DFI, showed no significant differences. These results suggest that poor male sleep quality is primarily associated with reduced semen quality rather than alterations in hormone profiles.

### Regression analysis between male sleep quality and semen parameters, as well as hormone profiles

The associations between male sleep quality and male reproductive parameters were assessed using multiple linear regression, with results presented in Table 4. Poor male sleep quality, as indicated by higher PSQI scores, was significantly associated with reduced sperm concentration (adjusted β = –1.39; 95% CI: –2.11 to –0.66; *P* < 0.001), PR (adjusted β = –1.27; 95% CI: –1.64 to –0.90; *P* < 0.001), and total motility (adjusted β = –1.30; 95% CI: –1.70 to –0.90;* P* < 0.001). No significant associations were found between total PSQI scores and hormone profiles, including FSH, LH, E2, PRL, or testosterone (all *P* > 0.05). Among individual PSQI components, subjective sleep quality, subjective sleep quality, habitual sleep efficiency, and sleep disturbances had the most notable negative effects on sperm quality. Daytime dysfunction was significantly related to decreased NM (adjusted β = –0.37; 95% CI: –0.64 to –0.09; *P* = 0.009). Although PRL was not generally affected, it showed a positive association with the use of sleeping medication in one model (adjusted β = 2.68; 95% CI: 0.54 to 4.82; *P* = 0.014). No significant associations were found between male sleep quality and DFI. These findings suggest that poor male sleep quality, especially in aspects related to perceived male sleep quality, efficiency, and disturbances, adversely affects sperm concentration and motility, with limited impact on hormone profiles.

**Table 4:** Multiple linear regression analysis of the association between the sleep quality and sperm parameters, reproductive hormones

	PSQI		PSQI		Subjective sleep quality		Habitual sleep efficiency		Sleep disturbances		Use of sleeping medication		Daytime dysfunction	
	β (95%CI)	P	Adjusted β (95%CI)	P	Adjusted β (95%CI)	P	Adjusted β (95%CI)	P	Adjusted β (95%CI)	P	Adjusted β (95%CI)	P	Adjusted β (95%CI)	P
FSH (mIU/mL)	0.03 (−0.07 ~ 0.12)	0.591	0.02 (−0.08 ~ 0.12)	0.714	−0.18 (−0.60 ~ 0.24)	0.403	−0.14 (−0.44 ~ 0.16)	0.374	0.13 (−0.38 ~ 0.63)	0.62	−0.02 (−0.49 ~ 0.46)	0.95	−0.08 (−0.40 ~ 0.24)	0.628
LH (mIU/mL)	−0.01 (−0.08 ~ 0.06)	0.852	−0.01 (−0.08 ~ 0.06)	0.707	−0.20 (−0.51 ~ 0.10)	0.189	−0.22 (−0.44 ~ −0.01)	0.047	−0.18 (−0.55 ~ 0.18)	0.33	0.06 (−0.29 ~ 0.41)	0.735	−0.05 (−0.29 ~ 0.18)	0.657
E2 (pg/mL)	−0.13 (−0.50 ~ 0.25)	0.515	−0.14 (−0.52 ~ 0.24)	0.469	−0.83 (−2.47 ~ 0.81)	0.322	−0.41 (−1.57 ~ 0.75)	0.487	−0.25 (−2.20 ~ 1.70)	0.804	1.05 (−0.81 ~ 2.92)	0.269	0.16 (−1.11 ~ 1.43)	0.802
PRL (ng/mL)	−0.15 (−0.58 ~ 0.29)	0.506	−0.14 (−0.58 ~ 0.30)	0.525	1.38 (−0.51 ~ 3.27)	0.153	−0.44 (−1.80 ~ 0.91)	0.523	1.54 (−0.73 ~ 3.81)	0.184	2.68 (0.54 ~ 4.82)	0.014	1.21 (−0.23 ~ 2.66)	0.101
T(ng/mL)	0.01 (−0.03 ~ 0.06)	0.505	−0.00 (−0.04 ~ 0.04)	0.895	0.07 (−0.09 ~ 0.23)	0.387	−0.02 (−0.13 ~ 0.10)	0.771	−0.03 (−0.23 ~ 0.16)	0.725	0.14 (−0.04 ~ 0.32)	0.139	0.05 (−0.07 ~ 0.17)	0.425
Concentration (10^6^/mL)	−1.39 (−2.11 ~ −0.67)	<.001	−1.39 (−2.11 ~ −0.66)	<.001	−5.79 (−8.91 ~ −2.67)	<.001	−2.16 (−4.41 ~ 0.10)	0.061	−3.78 (−7.55 ~ −0.01)	0.05	−2.03 (−5.60 ~ 1.54)	0.266	−3.38 (−5.79 ~ −0.98)	0.006
PR%	−1.25 (−1.61 ~ −0.88)	<.001	1.27 (−1.64 ~ −0.90)	<.001	−3.52 (−5.13 ~ −1.92)	<.001	−2.10 (−3.25 ~ −0.94)	<.001	−2.24 (−4.19 ~ −0.29)	0.024	−1.56 (−3.40 ~ 0.29)	0.099	−1.87 (−3.11 ~ −0.63)	0.003
NP%	−0.01 (−0.11 ~ 0.09)	0.837	−0.01 (−0.11 ~ 0.09)	0.845	−0.19 (−0.63 ~ 0.24)	0.381	0.03 (−0.28 ~ 0.34)	0.853	−0.21 (−0.73 ~ 0.31)	0.435	0.16 (−0.34 ~ 0.65)	0.534	−0.21 (−0.54 ~ 0.13)	0.224
Total activity%	−1.29 (−1.69 ~ −0.89)	<.001	−1.30 (−1.70 ~ −0.90)	<.001	−3.53 (−5.29 ~ −1.77)	<.001	−1.97 (−3.24 ~ −0.70)	0.002	−2.56 (−4.69 ~ −0.43)	0.019	−1.40 (−3.42 ~ 0.62)	0.176	−1.96 (−3.32 ~ −0.61)	0.005
NM%	0.08 (−0.16 ~ 0.00)	0.056	0.08 (−0.16 ~ 0.00)	0.056	0.03 (−0.33 ~ 0.39)	0.871	−0.15 (−0.40 ~ 0.11)	0.256	−0.08 (−0.51 ~ 0.35)	0.726	−0.09 (−0.50 ~ 0.31)	0.654	−0.37 (−0.64 ~ −0.09)	0.009
DFI%	−0.02 (−0.17 ~ 0.13)	0.768	−0.01 (−0.17 ~ 0.14)	0.859	0.22 (−0.41 ~ 0.86)	0.49	−0.12 (−0.58 ~ 0.34)	0.617	−0.00 (−0.76 ~ 0.76)	0.995	−0.13 (−0.86 ~ 0.61)	0.732	0.11 (−0.39 ~ 0.60)	0.672

Regression coefficients were adjusted for male age, male body mass index and male smoking status

*DFI* DNA fragmentation index, *E2* estradiol, *FSH* follicle-stimulating hormone, *LH* luteinizing hormone, *NM* sperm morphology, *NP* non-progressive motility, *PR* progressive motility, *PRL* prolactin, *T* testosterone

## Discussion

This study is the first to investigate the relationship between male sleep quality and male infertility, as well as the association between male sleep quality and pregnancy outcomes in China, thereby addressing a gap in the existing literature. Our findings indicate that different dimensions of sleep quality play varying roles in male infertility and pregnancy outcomes, highlighting the substantial impact of sleep health on male reproductive function and assisted reproductive success.

One key mechanism by which sleep disturbance may contribute to infertility is endocrine disruption. Testosterone is a crucial hormone for male fertility, directly affecting sperm production, maturation, and function. Poor male sleep quality, particularly the lack of rapid eye movement sleep and slow-wave sleep stages, may lead to a decline in testosterone levels. Studies have shown that chronic sleep deprivation or irregular sleep patterns significantly reduce testosterone levels, thereby affecting male sexual function and fertility [24, 25]. LH primarily stimulates Leydig cells to produce testosterone, which then diffuses into the seminiferous tubules to support spermatogenesis. Prolactin, on the other hand, increases the number of LH receptors, allowing LH to act on Leydig cells to enhance testosterone synthesis [26]. Our study found that while the PSQI total score had no significant impact on hormone levels, habitual sleep efficiency affected LH levels, and use of sleeping medication influenced PRL secretion. These findings suggest that sufficient and high-quality sleep helps maintain normal hormone levels, thereby supporting reproductive health. In addition to hormonal regulation and sperm quality, sleep deprivation also impairs male reproductive health by increasing oxidative stress.

Beyond hormonal pathways, oxidative stress offers another plausible explanation for impaired sperm quality in infertile men with poor sleep. Oxidative strain denotes a disruption in equilibrium between reactive oxygen species and defensive antioxidant mechanisms within the body, where excessive free radicals cause cellular damage [27]. Studies have demonstrated that insufficient sleep elevates oxidative stress levels, thereby compromising the health of reproductive system tissues, such as the testes and sperm [28, 29]. Our findings reveal a strong correlation between sleep efficiency and sperm health: as sleep patterns deteriorated, sperm concentration, progressive motility, and total motility all declined.. The observed reductions in sperm concentration and motility among men with poor sleep quality, although modest in absolute terms, are clinically relevant because even small impairments in these parameters can significantly affect fecundability and reproduction success rates. Although total sperm count and total motile sperm count are considered informative indicators of male fertility potential [30], we were unable to calculate these metrics due to inconsistent documentation of semen volume in our dataset. Additionally, the incremental predictive value of these parameters over standard WHO semen parameters is uncertain. Future prospective studies with standardized semen volume recording and broader inclusion of natural conception would allow these measures to be incorporated and their relevance further evaluated. Although no statistically significant difference was observed in DFI between groups, the role of sperm DNA integrity in fertilization and embryo development warrants consideration; thus, these findings should be interpreted within the broader context of oxidative stress and genomic stability in sperm. Systemic hormones (testosterone, LH, FSH, prolactin) did not differ by male sleep quality in our cohort, and poorer sleep still correlated with worse semen parameters. This suggests that local testicular oxidative stress, rather than endocrine disruption, may drive sperm damage: the testes’ high mitochondrial activity and rapid cell division during spermatogenesis make them especially vulnerable to reactive oxygen species (ROS)-induced lipid peroxidation and DNA fragmentation. Future work should therefore assess seminal ROS levels, testicular antioxidant enzyme activity, and markers of lipid peroxidation to clarify how sleep loss impairs fertility despite normal serum hormones.

Importantly, our data also reveal a link between male sleep quality and pregnancy outcomes in couples attempting to conceive naturally. Men with PSQI scores above the clinical threshold were less likely to achieve pregnancy, suggesting that male sleep disturbance may reduce fecundity. Poor sleep may impair sperm health through both endocrine and oxidative pathways, and it may also influence couple dynamics such as timing and frequency of intercourse that affect conception. Future research should examine seminal ROS markers, testicular antioxidant capacity, and behavioral factors to clarify how sleep interventions could improve both semen quality and live birth rates.

This study has several strengths. It employed the internationally standardized PSQI scale to assess male sleep quality, enhancing the reliability and comparability of the findings. By focusing on infertile couples in China, the study offers a comprehensive analysis of male sleep quality and its potential impact on pregnancy outcomes, thereby expanding the scope of research in male reproductive health. From a clinical perspective, our findings suggest potential interventional opportunities, such as behavioral sleep therapy, cognitive-behavioral interventions for insomnia, and melatonin supplementation, which could be evaluated for their effects on male fertility. Future studies should also investigate downstream reproductive outcomes, including live birth rates, circadian hormonal fluctuations, and oxidative stress biomarkers, to elucidate mechanistic pathways and optimize intervention strategies.

### Study limitations

Several limitations should be acknowledged. The single-center design and exclusive focus on a Chinese population may limit the generalizability of the results to other settings or cultural contexts. Potential confounders, such as psychological status, lifestyle factors, and the female partner’s sleep quality, were not fully controlled, which may have influenced the observed associations. In addition, important potential confounders such as the female partner’s sleep quality, psychosocial stress, and occupational work schedules were not assessed in the present study. These unmeasured variables may influence both male sleep patterns and reproductive outcomes, and should be considered in future investigations. The reliance on self-reported sleep data introduces the possibility of recall bias, and the PSQI's one-month assessment window may not fully capture long-term sleep patterns relevant to fertility. Consequently, the temporal relationship between habitual sleep and reproductive outcomes could not be fully assessed. It is also possible that reverse causation may partly explain our findings, whereby infertility-related stress or lifestyle adjustments could adversely impact male sleep quality. This bidirectional relationship underscores the need for longitudinal designs to disentangle cause and effect.Future studies should adopt multicenter designs, larger and more diverse populations, better control for confounding variables, and incorporate longer follow-up periods to validate and extend these findings.

## Conclusion

Poor male sleep quality is significantly associated with impaired semen quality and reduced pregnancy success in infertile couples. These results highlight the potential clinical value of incorporating sleep assessment and management into male infertility evaluation. Interventions aimed at improving sleep quality such as behavioral sleep therapy, cognitive-behavioral interventions for insomnia, or circadian rhythm optimization, may represent an accessible and non-invasive approach to enhance male reproductive health and fertility outcomes. Future multicenter, longitudinal studies are warranted to confirm these associations and to determine whether improving sleep quality can translate into higher natural conception and live birth rates.

## Supplementary Information

Supplementary Material 1.

## Data Availability

Data are available from the corresponding author upon reasonable request.

## Abbreviations

BMI: Body mass index
CI: Confidence interval
DFI: DNA fragmentation index
E2: Estradiol
FSH: Follicle-stimulating hormone
LH: Luteinizing hormone
NP: Non-progressive motility
OR: Odds ratio
PR: Progressive motility
PRL: Prolactin
PSQI: Pittsburgh Sleep Quality Index
ROS: Reactive oxygen species
SD: Standard deviation
T: Testosterone
