# Supplementary material for: Association of male sleep quality with semen parameters and pregnancy outcomes in infertile couple
Source: Basic Clin Androl. 2025 Oct 1;35:37. doi: 10.1186/s12610-025-00287-w (PMC12487260; doi:10.1186/s12610-025-00287-w)
Supplement: Supplementary file 1 — Supplementary Material 1. [file 12610_2025_287_MOESM1_ESM.docx]

**Supplementary materials**

**1. The Pittsburgh Sleep Quality Index (PSQI)**

Instructions: The following questions ask about your sleep habits during the past month. Please answer each question as accurately as possible. If you are not sure about a specific question, answer it as best you can.

1. Subjective Sleep Quality

1. During the past month, how would you rate your sleep quality overall?

- 0 = Very good
- 1 = Fairly good
- 2 = Fairly bad
- 3 = Very bad

2. Sleep Latency

2. During the past month, how long (in minutes) has it taken you to fall asleep each night?

- 0 = 0–15 minutes
- 1 = 16–30 minutes
- 2 = 31–60 minutes
- 3 = 61–90 minutes
- 4 = More than 90 minutes

3. Sleep Duration

3. During the past month, how many hours of actual sleep did you get at night?

- 0 = More than 7 hours
- 1 = 6–7 hours
- 2 = 5–6 hours
- 3 = 4–5 hours
- 4 = Less than 4 hours

4. Sleep Efficiency

4. During the past month, how often have you had trouble staying awake and alert during the daytime?

- 0 = No trouble
- 1 = A little trouble
- 2 = Some trouble
- 3 = A lot of trouble

(There is also a calculation method here for sleep efficiency that involves dividing actual sleep time by total time spent in bed, but this is usually done during scoring.)

5. Sleep Disturbances

5. During the past month, how often have you had trouble sleeping due to the following factors?

(For each item, rate from 0 = never, to 3 = very frequent)

- a. Noise (e.g., snoring, traffic)
- b. Breathing difficulties (e.g., shortness of breath, apnea)
- c. Pain (e.g., back pain, headache)
- d. Feeling too hot or cold
- e. Having to get up to use the bathroom
- f. Coughing or other symptoms
- g. Bad dreams or nightmares
- h. Other factors (specify)

6. Use of Sleeping Medication

6. During the past month, how often have you used medication (prescription or over-the-counter) to help you sleep?

- 0 = Never
- 1 = Less than once a week
- 2 = Once or twice a week
- 3 = Three or more times a week

7. Daytime Dysfunction

7. During the past month, how much of a problem has it been for you to stay awake and alert during daytime activities?

- 0 = No problem
- 1 = A slight problem
- 2 = A moderate problem
- 3 = A severe problem

Scoring Instructions:

- The Total PSQI Score is obtained by summing the scores for the seven components.
- Total Score Range: 0 to 21
  - A score greater than 5 indicates poor sleep quality.
